# Patients with rare diseases are responsible for the majority of hospitalizations in a Brazilian tertiary pediatric hospital: Preliminary data

**DOI:** 10.1016/j.clinsp.2024.100535

**Published:** 2024-11-22

**Authors:** Cristina Grassiotto, Simone Pavani, Nara Vasconcelos Cavalcanti, Angelina Gonçalves, Andreia Watanabe, Vicente Odone-Filho, Ana Cristina Aoun Tannuri, Clovis Artur A. Silva, Magda Carneiro-Sampaio

**Affiliations:** Instituto da Criança, Hospital das Clínicas, Faculdade de Medicina, Universidade de São Paulo, São Paulo, SP, Brazil

As 2024 is a leap year, the 29th of February represents a special opportunity to draw attention to the so-called Rare Diseases (RDs), although every year the last day of this month has been dedicated to this group of conditions. RDs comprise an already large and rapidly growing category of diseases frequently associated with a chronic course, representing a pressing challenge for broad and adequate patient care in all countries, particularly in developing ones.

There is still no international consensus on the definition of RD. The Brazilian Ministry of Health adopts the WHO concept, that is, a disease with a frequency ≤ 65:100,000 inhabitants or 1.3:2000.^1^ These numbers are quite similar to those adopted by European Union countries, which correspond to ≤1:2000 inhabitants. It is estimated that >80 % of RDs have genetic etiology, currently distributed among 7000–8000 different described entities.^2^ The frequency of RDs as a group in the general population is estimated at 5–6 %, however, there is recent evidence that these numbers may be higher and even reach 10 %. Therefore, we may have 10–12 million people with RDs in our country, with a fifth of them living in the State of São Paulo.

Preparing for Rare Disease Day, between January 29th and February 2nd, 2024, the diagnoses of all infants, children, and adolescents admitted to the wards and intensive care units of the HCFMUSP Children's Hospital (“Instituto da Criança e do Adolescente” – ICr) were compiled and each patient was classified as having a RD or not according to the frequency of the disease as per medical literature. We had to refer particularly to international data due to the lack of Brazilian literature in this field.

A total of 128 patients were admitted to the 8 wards and intensive care units located in the aforementioned tertiary hospital in those five-week days of observation. Among them, 60 % (77/128) had a confirmed diagnosis of a RD. Additionally, 5.5 % (7/128) of the patients had a chronic disease still under etiological investigation, which once confirmed could increase even more the proportion of RDs.

The most frequent group of diseases was rare cancers (14 patients), represented by: neuroblastoma, hepatoblastoma, Wilms tumor, round cell sarcoma, ependymoma, pheochromocytoma, osteosarcoma, Burkitt's lymphoma and desmoid tumor. The second most common group of RDs was composed of patients with chronic kidney diseases (13 patients), whose diagnoses were: Prune Belly syndrome (*n* = 4), autosomal recessive polycystic kidney disease (*n* = 3), posterior urethral valve (*n* = 3), nephropathic cystinosis (*n* = 2), and atypical hemolytic uremic syndrome (*n* = 1), and 4 of them had already undergone organ transplantation as a consequence of the specific RD. The third largest group (12 patients) was represented by malformations of the gastrointestinal tract, defects of the abdominal wall (gastroschisis), and diaphragmatic hernias, all requiring surgical interventions, which occurred more frequently in the neonatal period. It is noteworthy that around 80 % of the infants in the neonatal intensive care unit of this hospital presented distinct surgical conditions. Of note, patients with congenital heart defects are treated separately at the Heart Institute (Instituto do Coração ‒ InCor), also part of the same academic medical center complex.

Additionally, other 10 patients had chronic liver diseases with 8 of them requiring transplantation, and the underlying conditions were biliary atresia, progressive familial intrahepatic cholestasis type 3, alpha1-antitrypsin deficiency, and autoimmune hepatitis, also RDs. Other diagnoses observed included: malformations of the central nervous system (5), autoimmune diseases (3), cystic fibrosis (2), inborn errors of immunity (2), hematologic diseases (2), inborn errors of metabolism (2), among others.

This type of periodic survey, conducted in moments with no seasonal outbreaks, can be useful to identify the disease profile of our hospitalized patients and, thus, provide them with better care. On the other hand, it is difficult to compare our data with those of other tertiary pediatric hospitals due to the peculiarities of our Institution, which receives the most complex and severe patients within a hierarchical system organized to provide comprehensive care to the population. Although rarely included in the RDs groups, congenital malformations of the digestive tract, abdominal wall defects, and diaphragmatic hernias ‒ the third most common category in this survey ‒ all fall into this classification. These patients require more attention for early detection, largely during prenatal care in most conditions, and referral to a pediatric surgical service capable of offering adequate and prompt treatment for these newborns. In most cases, the malformation is an isolated defect, and these children may have a good prognosis without a long-term burden to the healthcare system.

Furthermore, our data indicate that RDs require more attention in our healthcare system, and the availability of advanced genetic testing is a critical aspect in this regard. Indeed, a survey conducted at our Children's Hospital found that delays in establishing the diagnosis and difficulties in accessing specialized services and next-generation DNA sequencing testing are the biggest challenges reported by families and physicians alike.^3^

Aiming at providing early diagnosis and improving the assistance to patients with genetic diseases, as well as stimulating research and teaching in this area, our academic medical complex launched in 2023 the Integrated Center for Genetic Diseases (Centro Integrado de Doenças Genéticas – CIGEN https://www.fm.usp.br/fmusp/centros-interdepartamentais/centro-integrado-de-doencas-geneticas-cigen). This is a new and unique collaborative network of diagnostic services, medical assistance, rehabilitation and research laboratories. Several Departments in our academic medical center are already involved in CIGEN: Cardio-Pneumology, Dermatology, Internal Medicine, Neurology, Obstetrics & Gynecology, Oncology, Ophthalmology & Otorhinolaryngology, Orthopedics & Traumatology, Pathology, Pediatrics, Physical Medicine & Rehabilitation, Preventive Medicine, Psychiatry, and Radiology. All working in collaboration to provide better care to our patients.

## Declaration of competing interest

The authors declare no conflicts of interest.
